# Development of a Web-Based Interactive Tool for Visualizing Breast Cancer Clinical Trial Tolerability Data

**DOI:** 10.1200/CCI.24.00007

**Published:** 2024-07-16

**Authors:** Michael Luu, Gillian Gresham, Lynn Henry, Sungjin Kim, Andre Rogatko, Greg Yothers, Ron D. Hays, Mourad Tighiouart, Patricia A. Ganz

**Affiliations:** ^1^ Cedars Sinai Medical Center, Department of Computational Biomedicine, Los Angeles, CA; ^2^ University of Michigan Rogel, Cancer Center, Ann Arbor, MI; ^3^ Department of Biostatistics, University of Pittsburgh, Pittsburgh, PA; ^4^ University of California Los Angeles, Department of Medicine, Los Angeles, CA; ^5^ University of California Los Angeles, Jonsson Comprehensive Cancer Center, Los Angeles, CA

## Abstract

**PURPOSE:**

Longitudinal patient tolerability data collected as part of randomized controlled trials are often summarized in a way that loses information and does not capture the treatment experience. To address this, we developed an interactive web application to empower clinicians and researchers to explore and visualize patient tolerability data.

**METHODS:**

We used adverse event (AE) data (Common Terminology Criteria for Adverse Events) and patient-reported outcomes (PROs) from the NSABP-B35 phase III clinical trial, which compared anastrozole with tamoxifen for breast cancer–free survival, to demonstrate the tools. An interactive web application was developed using R and the Shiny web application framework that generates Sankey diagrams to visualize AEs and PROs using four tools: AE Explorer, PRO Explorer, Cohort Explorer, and Custom Explorer.

**RESULTS:**

To illustrate how users can use the interactive tool, examples for each of the four applications are presented using data from the NSABP-B35 phase III trial and the NSABP-B30 trial for the Custom Explorer. In the AE and PRO explorers, users can select AEs or PROs to visualize within specified time periods and compare across treatments. In the cohort explorer, users can select a subset of patients with a specific symptom, severity, and treatment received to visualize the trajectory over time within a specified time interval. With the custom explorer, users can upload and visualize structured longitudinal toxicity and tolerability data.

**CONCLUSION:**

We have created an interactive web application and tool for clinicians and researchers to explore and visualize clinical trial tolerability data. This adaptable tool can be extended for other clinical trial data visualization and incorporated into future patient-clinician interactions regarding treatment decisions.

## Teaser

Interactive web app for clinicians to explore & visualize patient tolerability data in clinical trials

## INTRODUCTION

The National Cancer Institute's Patient-Reported Outcomes version of the Common Terminology Criteria for Adverse Events (PRO-CTCAE) Measurement System has stimulated the collection of patient-reported toxicity data.^1^ The US Food and Drug Administration (FDA) launched an initiative called Project Patient Voice (PPV) within its Oncology Center of Excellence^2^ to make adverse events (AEs) and PRO data from cancer clinical trials more accessible to patients, researchers, and health care providers. There have been increasing efforts to improve methods for summarizing cancer treatment tolerability data with the use of toxicity scoring and summary measures, such as maximum grade, composite grade,^3^ and the toxicity index (TI).^4^ Much less has been done to develop better visualization of longitudinal AE and PRO data for patients, clinicians, and researchers.^5^ With the increased availability of information on treatment tolerability, there is a greater need for new methods of visualizing these data.

**CONTEXT**

**Key Objective**
To develop a web application to explore clinical trial tolerability data for clinicians and researchers.
**Knowledge Generated**
This novel tool visualizes the existing trial data (toxicity and symptoms) and allows uploading of new data. Users can personalize their experience and explore symptom trajectories.
**Relevance (*J.L. Warner*)**
Tools such as this offer the possibility of bringing data to patients and providers in a intuitive format. Generalizability to other trial data settings should be explored next.*
*Relevance section written by *JCO CCI* Editor-in-Chief Jeremy L. Warner, MD, MS, FAMIA, FASCO.

In this article, we describe the development of various publicly available web-based interactive applications that use R and the Shiny web application frameworks to visualize treatment toxicity and PRO data over time in patients with cancer. PPV is moving in this direction by providing static visualizations of data (eg, max grade or PRO-CTCAE) such as bar plots and pie charts. The suite of tools developed, as detailed in this article, aims to enhance PPV's efforts in enabling researchers, clinicians, and patients to interactively explore data. Using data from a breast cancer clinical trial, we demonstrate the application's functionality in summarizing AEs and PROs through various visualization tools such as text, bar graphs, and Sankey diagrams.

## METHODS

### Data Source

We used data from the NSABP-B35 phase III randomized clinical trial that compared anastrozole with tamoxifen in postmenopausal women with ER-positive ductal carcinoma in situ, which was accessed and used as part of our U01 Cancer Moonshot grant.^6^ The NSABP-B35 trial was double-blind, where patients were randomly assigned on a 1:1 basis to either receive anastrozole or tamoxifen within 30 days of random assignment with the end of treatment 5 years from the date of first dose.

Items from the Breast Cancer Prevention Trial (BCPT) symptom checklist were used to collect symptom severity data before treatment and every 6 months for 5 years. Patients were clinically assessed every 6 months while on study, with CTCAE ratings by the treating clinician at those time points for the first 60 months and then every 12 months afterward until death or loss to follow-up.

The NSABP-B30 clinical trial^7^ randomly assigned patients with early-stage breast cancer to three different adjuvant chemotherapy regimens: doxorubicin (A) and cyclophosphamide (C) followed by docetaxel (T; sequential ACT); doxorubicin and docetaxel (AT); or doxorubicin, cyclophosphamide, and docetaxel (concurrent ACT). Items from the BCPT symptom checklist were collected at pretreatment baseline, day 1 of cycle 4, and every 6 months up to 2 years from baseline for all three treatment arms. Additional details of the trial have been described elsewhere.^7^ Patient data from both trials were deidentified and shared following the institutional and NRG oncology institutional review board (IRB) standards.

### Development

Four web-based applications were developed to summarize AE data associated with cancer clinical trials: AE Explorer, PRO Explorer, Cohort Explorer, and Custom Explorer. All the applications were developed using R and the shiny web application framework.^8,9^ The web-based applications are hosted on shinyapps.io by Posit, and all components of this application are freely available as open-source software.^10^ Each application features an interactive menu, allowing users to select scenarios by AEs, symptom, severity, and time point. The selection of these scenarios varies by application, as elaborated further in the description of the tools below.

### Sankey Diagrams

Sankey diagrams, named after Irish captain Matthew Riall Sankey, were used to visualize longitudinal toxicity data.^11^ There are three primary parts of the Sankey diagram: the Node, Flow, and Stage (Data Supplement, Fig S1). We modeled each of these parts of the diagram in the context of a clinical trial assessing symptoms across various cycles or time points of a patient's treatment regimen. The Nodes are depicted as the patient's symptom severity grouped in Stages or the patient's treatment cycle. The height of each node depicts the relative number of patients with a given grade within a given cycle. The Flow or colored bands depict the flow of patients within each grade and between each treatment cycle.

The primary Sankey diagram is visualized using the package ggsankey developed by David Sjoberg.^12^ This package provides an extension for the underlying ggplot2 R package developed by Hadley Wickham^13^ from Posit to create Sankey diagrams.

### TI

The TI was used to summarize the CTCAE and PRO data for visualizations in the AE explorer tool. TI is a measure developed by Rogatko et al^14^ which summarizes the patient's overall toxicity profile (range, 0 to <6) into a single measure of overall severity (see the Data Supplement for a detailed definition). Its statistical properties have been described by Razaee et al.^15^ It has been further validated in additional analyses including CTCAE data from the NSABP R-04,^4^ NSABP B-30,^16^ and NSABP B-35^17^ trials and applied to PRO data.^18^

### Applications

The development of each of the four applications is described below. The tools can be accessed at GitHub.^19^

#### PRO Cohort Explorer

The PRO Cohort Explorer enables the exploration of a specific patient cohort using BCPT symptom checklist data from the NSABP-B35 trial. The user inputs allow selection of the specific patient cohort of interest to explore. Users can define their cohort by selecting a treatment of interest (Anastrozole or Tamoxifen), the specific BCPT symptom, the cohort's initial symptom severity level (Not at all, Slightly, Moderately, Quite a bit, Extremely, and No Response) for the selected symptom, and the initial and final time points of interest.

The application generates a Sankey diagram and summary statement detailing patient responses at initial and final time points. Nodes in the Sankey diagrams are color-coded to represent symptom severity (Not at all, Slightly, Moderately, Quite a bit, Extremely, and No Response). The Sankey stages are displayed in 6-month intervals over a specified time interval, as selected by the user.

#### AE Cohort Explorer

Similar to the PRO Cohort Explorer, the AE Cohort Explorer focuses on specific patient cohorts within the NSABP-B35 clinical trial using CTCAE data. The tool shares similar inputs: users select: Treatment (Anastrozole, Tamoxifen), followed by CTCAE symptom term, cohort severity grade at the initial time point (grades 0-1, 2, 3, 4, 5), and initial and final time points.

Results include separate tabs for Sankey Diagram, Grade Duration, and TI. The Sankey Diagram tab mirrors the AE Explorer output with stages presented in 6-month intervals. The Grade Duration tab features a bar graph showing patient grades at each time point. The TI tab displays a histogram distribution of the TI within the selected patient cohort, with summary statistics such as mean (standard deviation) and median (IQR).

#### AE Explorer

The AE Explorer application allows the user to explore the CTCAE data from the NSABP-B35 clinical trial. This application allows the user to generate Sankey diagrams for patient cohorts stratified by treatments and AEs. Within this application, multiple AEs may be consecutively selected for visualization.

The AE Explorer constructs separate Sankey diagrams for each treatment and AEs that are selected. The nodes are color-coded to indicate symptom severity, on the basis of the CTCAE grading (grades 0-1, 2, 3, 5, Off Treatment). The stages are depicted as cycles in 6-month intervals from 6 months to 60 months. Additional visualizations, including bar plots of the TI, are also generated as part of this tool. Once visualized, the diagrams may be generated and exported into a downloadable PDF report.

#### Custom AE/PRO Explorer

The Custom AE/PRO Explorer mirrors the AE Cohort Explorer's structure; however, it allows users to upload structured clinical trials data into this module to construct Sankey diagrams. The input data must include three columns: patient identifier, time point, and symptom/response. The data should be in wide format, with the patient identifier repeated for each time point and symptom response. An example template of the data structure is included in the Data Supplement (Fig S2).

On first use, users are prompted to upload their clinical trial data to be visualized. Once uploaded, the user will need to define the column name pertaining to the patient identifier, time point, and finally the column containing the PRO response. Next, the time point and responses will need to be ordered. Once defined, the visualize button will generate the Sankey diagram on the basis of the defined parameters. Furthermore, the figure can be exported and downloaded as a PDF document.

## RESULTS

To illustrate how the interactive tool can be used for each of the four applications, we present four examples using the NSABP-B35 phase III clinical trial data.

### AE Cohort Explorer

A user may be interested in the trajectory of patients with grade 3 arthralgia at 6 months and the outcome of those patients at 30 months treated with anastrozole. AE Cohort Explorer produces various outputs that allow the user to further explore this cohort of patients.

**FIG 1.**
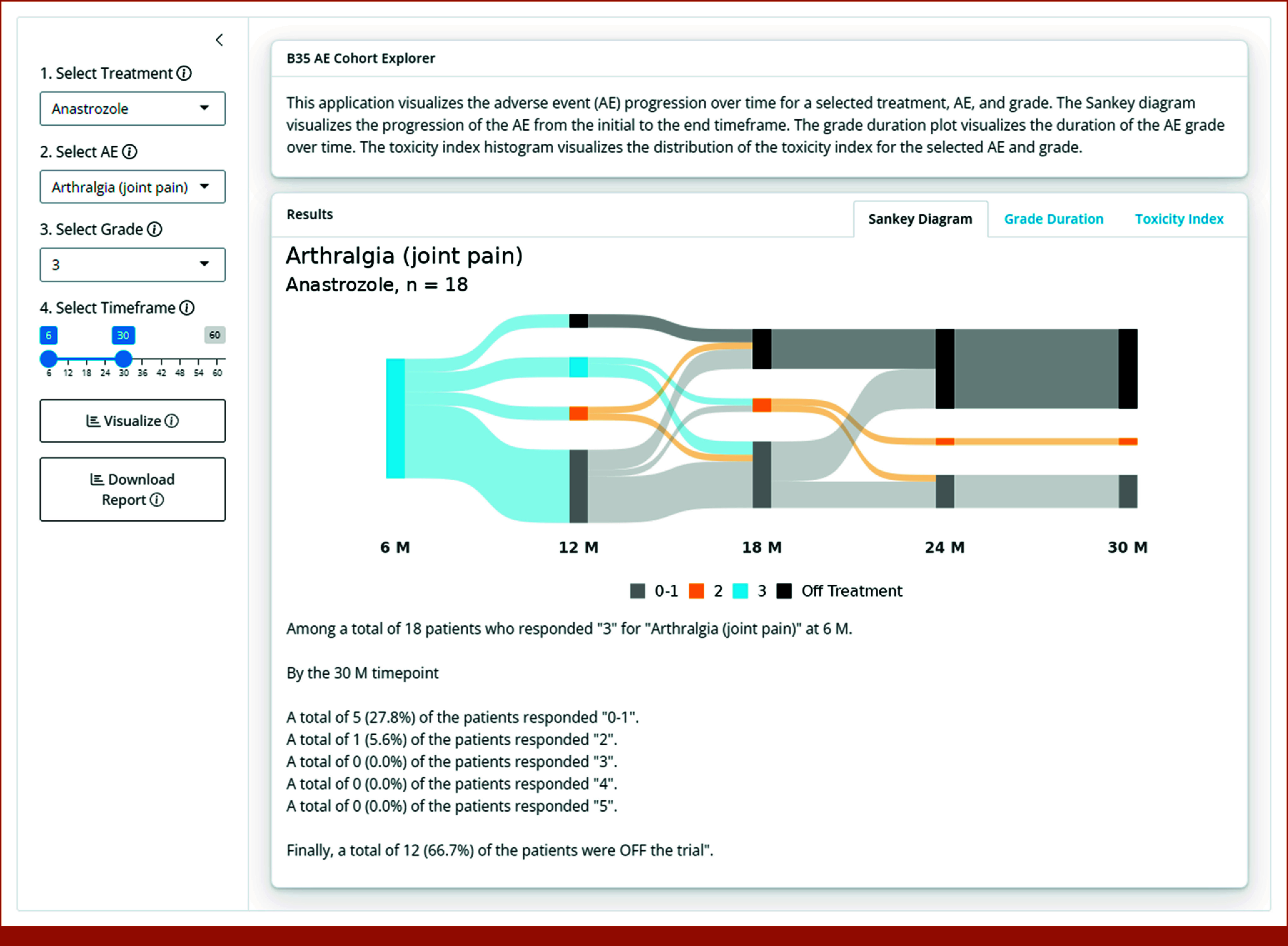
Example screenshot of the AE cohort explorer. AE, adverse event.

The Sankey Diagram tab produces a Sankey diagram of the patient cohort and a summary description of the outcome at the end cycle (30 months) as seen in Figure 1. There were 18 patients who exhibited Grade 3 arthralgia at 6 months treated with anastrozole. A large majority of patients were of grade 0-1 at 12 months, with a fewer number of patients who experienced grade 2 and grade 3 at cycle 2. A descriptive summary statement is generated for the final time point of interest (30 months; Fig 1). By the final cycle at 30 months, most of the patients (12% or 67%) were off trial, 5 of the initial 18 patients (28%) experienced grade 0-1 and 1 (6%) continued to experience grade 2.

**FIG 2.**
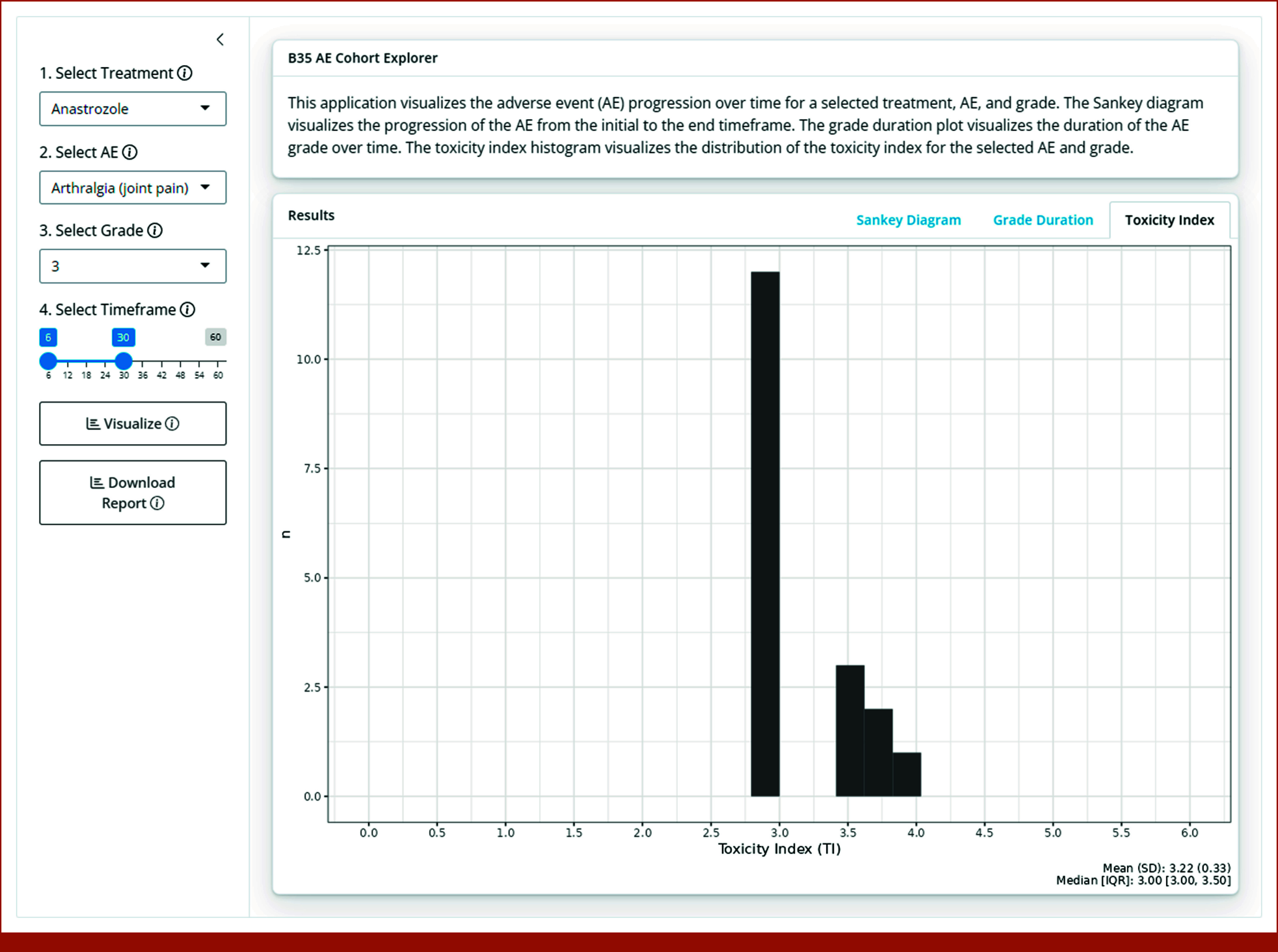
Example visualization of the toxicity index from the AE explorer. AE, adverse event.

The TI tab visualizes the TI distribution for the chosen cohort (grade 3, arthralgia, 6 months, anastrozole) using a histogram (Fig 2). The maximum grade was 3. Twelve patients never exceeded this grade, whereas the remaining received grades 2 and 3. The mean TI was 3.22 (0.33), and median TI was 3.0 [3.0-3.5].

The Grade Duration tab features a bar chart visualizing grade severity distribution by cycle. Users can explore grade prevalence over time. For example, 86% of grade 3 cases occurred at 6 months, with the remainder at 12 months. Similarly, grade 0-1 peaked at 12 (36%) and 18 months (32%), with lower proportions at 24 and 30 months (16%).

### PRO Cohort Explorer

A user may be interested in the trajectory of PROs, among patients treated with tamoxifen who exhibited moderate hot flashes from baseline (0 months) to 24 months. The PRO Cohort Explorer allows the user to explore the trajectory of responses using Sankey diagrams for the selected cohort of patients.

**FIG 3.**
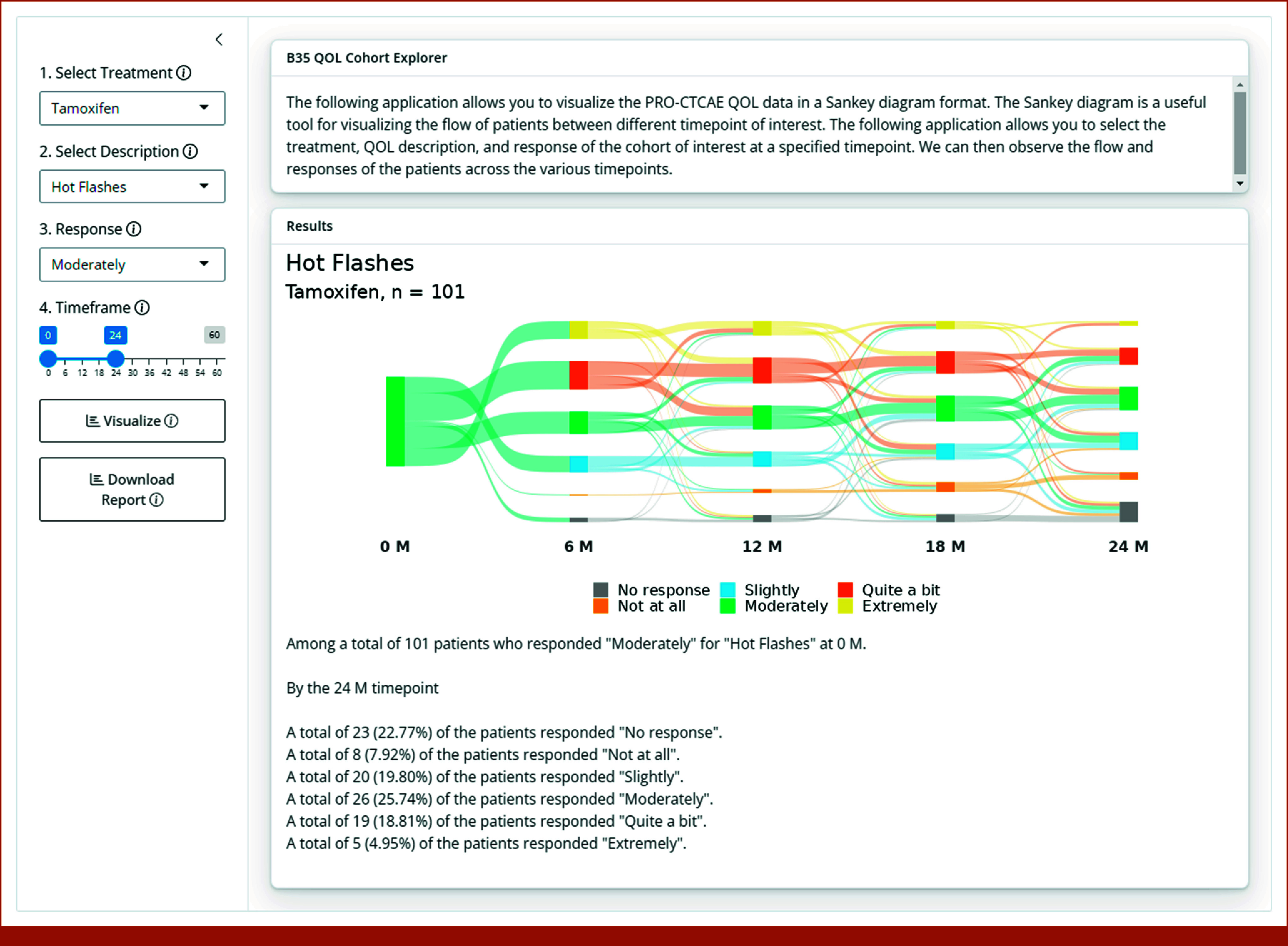
Example screenshot of the PRO cohort explorer. PRO, patient-reported outcome

There were a total of 101 patients who responded “Moderately” in the baseline form for hot flashes treated with tamoxifen (Fig 3). The Sankey diagram describes the flow of the selected cohort of patients who responded moderately at baseline to the responses at the final time point of 24 months. At 24 months, the majority of patients still responded moderately (26%) and slightly (20%), followed by quite a bit (19%) and extremely (5%). The remaining patients did not respond at all (8%) or had no response (23%).

### AE Explorer

Instead of exploring a specific cohort of patients, a user may be interested in exploring several CTCAE symptom terms across a set time interval. The AE explorer contains a single input that allows the user to select a number of CTCAE symptom terms. For example, the user can select Arthralgia (joint pain), Headache, and Hot flashes/flushes. The AE Explorer generates two Sankey diagrams for each CTCAE symptom term (separately for anastrozole and tamoxifen) visualizing the flow of patients from 6 months to 60 months.

### Custom AE/PRO Explorer

**FIG 4.**
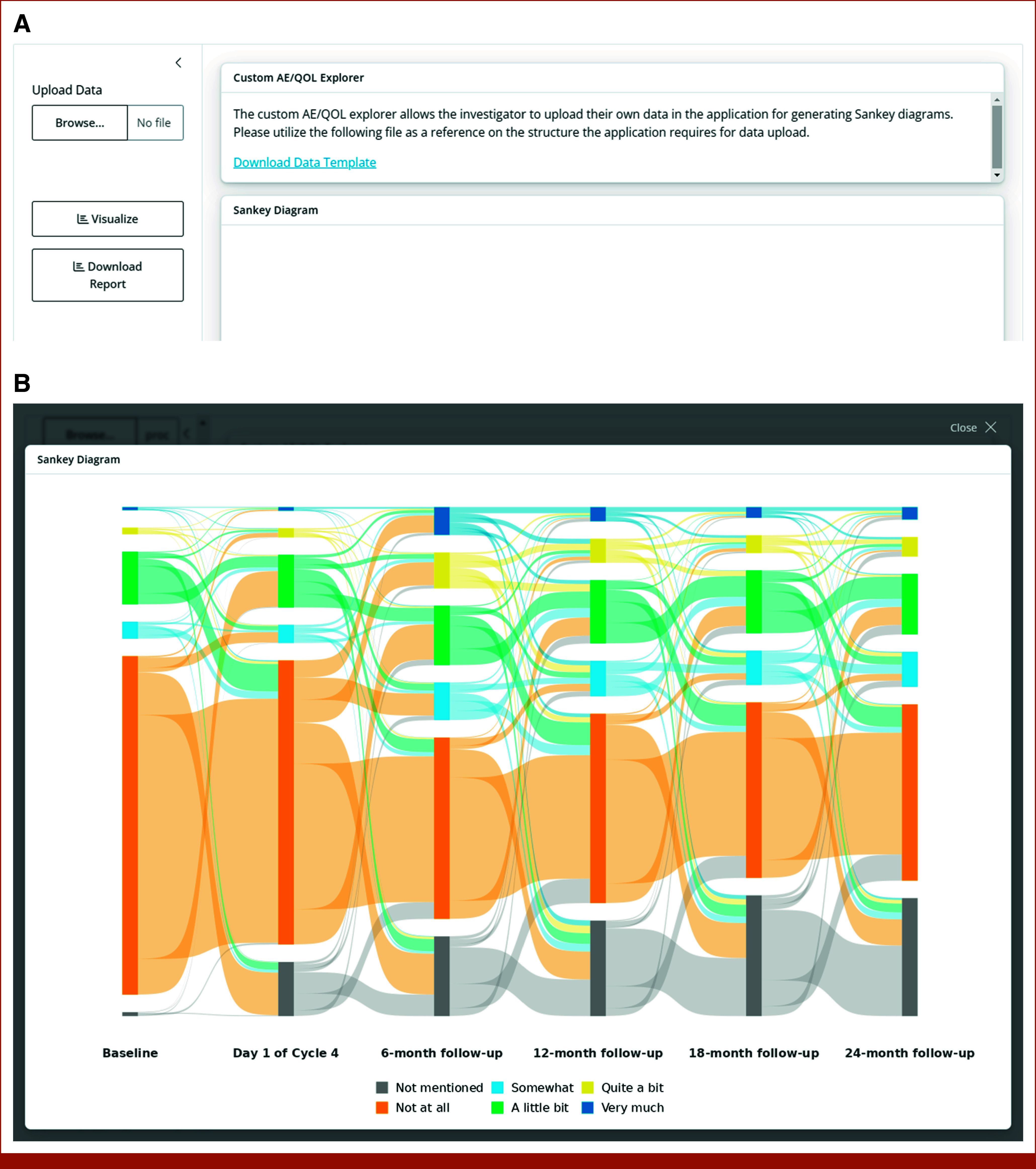
(A) Example interface of the custom explorer—uploading data. (B) Example output from the custom explorer: Sankey Diagram.

The user may be interested in exploring and visualizing data from their own or another clinical trial. As a case example, data from a different trial (B30) were uploaded to illustrate the tool's applicability to independent data sets. Figures 4A-4B show screenshots of the uploading process and output. Although additional clinical trials may be added as data become publicly available, it is the user's responsibility to use the Custom AE/PRO explorer to explore other clinical trial data.

We begin by uploading the processed data into the Custom AE/PRO explorer using the *upload data* tool. Once uploaded to the application, the user will define the column pertaining to the patient identifier (patient_id), time point (crs), and PRO/AE of interest (numb). Once selected, the user will define the ordering of the time point relevant to the trial to be visualized in the Sankey diagram (Baseline, day 1 of cycle 4, 6-month follow-up, 12-month follow-up, etc). We will next define the ordering of the response relevant to the trial (Not mentioned, Not at all, Somewhat, A little bit, Quite a bit, Very much). Once done, the *Visualize* button will generate a Sankey diagram on the basis of the defined parameters.

For this example using the B30 PRO data visualizing numbness and tingling (numb) from baseline to 12-month follow-up, a large proportion of patients responded “Not at all” during the baseline assessment. Among those reporting “Not at all” at baseline, the largest proportion of patients responded “Not at all” during day 1 of cycle 4, followed by “A little bit,” “Somewhat,” “Quite a bit,” and finally, “Very much.”

## DISCUSSION

We developed an interactive web application to explore and visualize clinical trial tolerability data for clinicians and researchers. In this article, we illustrate different applications within the tool to maximize the use of available longitudinal tolerability data, including toxicity and patient-reported symptom data, using two large clinical trials^6,7^ as case examples. To our knowledge, this is the first application of its kind that interactively allows for the visualization of the existing trial data and the uploading of data to be widely used by other researchers and clinicians.

The use of interactive tools can enhance patient understanding and engagement and improve the decision-making process between patients and providers.^20,21^ These tools can aid patients in anticipating treatment effects and visualizing trajectories over time. The FDA's PPV offers a similar platform, which allows providers to access patient-reported symptom data from clinical trials and use when communicating the benefits and risks of a particular treatment with their patients. PPV^2^ is currently in the pilot phase, using PRO-CTCAE data from the AURA3 trial of patients with advanced non–small cell lung cancer as an example. Visualizations, including bar graphs, pie charts, and tables, display patient symptom experiences by reported symptom, treatment, and over time.

Our tool enhances this concept, providing an interactive platform for users to select cohorts on the basis of symptoms, severity, initial time point, time intervals, and treatments, offering a personalized approach tailored to each of their unique experiences. Sankey diagrams in our tool help visualize symptom flow over time, maximizing the longitudinal nature of our data.^22^ Sankey plots are increasingly being recognized as effective visualizations to show the flow of symptoms from one state to another over time.^23^ Thus, they play an important role in the development of our web-based tool to provide an interactive and graphical display of the trajectory of symptoms to its users for each selected scenario. In addition, the Custom Explorer enables users to format and upload their own trial data to visualize AE or PRO data from any trial, as demonstrated with a separate data set. This feature will be important as it can help users summarize their own data and integrate them into their clinical practice.

While this tool was originally developed as part of the NCI Moonshot U01 (1U01CA232859-01) focusing on the analysis and visualization of toxicity data for clinicians and researchers, we added a patient-facing component for symptom visualization with the intent to share with providers. This parallel tool is currently under evaluation involving focus groups consisting of patients treated with breast cancer and the oncologist to refine and evaluate these tools. The study was IRB-approved (University of California Los Angeles, IRB No. 22-000395) with initial qualitative findings to be presented in a separate manuscript.

Some of the limitations and constraints of the tools include the necessary computational power and cost of maintaining and hosting a Shiny server. Data uniformity in clinical trials data also proved to be challenging as the tool was developed with a specific data structure in mind. The tool requires users to maintain data consistency in their submissions, for which we have provided detailed instructions and a sample template. As a web-based tool, it requires Wi-Fi and a device, which may pose accessibility and compatibility issues.^24,25^ To improve equity in digital health interventions, including older adults, strategies should be considered while disseminating the tool. Clinicians could address this by providing tablets with the tool in clinics for patient and clinician access. Third, the tool uses existing clinical trial data, which may be incomplete or of varying quality, leading to potential missing data and limited symptom information. Therefore, results should be interpreted cautiously and discussed with patients' oncologists. Finally, as this tool evolves, its design and features are continuously refined on the basis of user feedback, making it an ever-improving web-based resource.

Despite its limitations, the tool's user-friendly design, online presence, adaptability to various devices, and data upload capability enhance its feasibility and potential for widespread impact. It can be adapted to accommodate various PRO and toxicity data, new treatments, and future extensions for different trial designs.

In conclusion, we illustrate the potential and practicality of a web-based tool designed to encapsulate and visualize clinical trial tolerability and symptom data. This tool enables the translation of routinely collected longitudinal data into actionable insights for clinicians and their patients. In addition, we show how this tool can be extended by other researchers by incorporating their own clinical trial AE and PRO data into the Custom Explorer. Next steps for this research include further expanding the tools to include more clinical trial data, testing the usability and acceptability of the research and patient-facing tools in focus group settings, and evaluating the tools in clinical trials.
